# Has information on suicide methods provided via the Internet negatively impacted suicide rates?

**DOI:** 10.1371/journal.pone.0190136

**Published:** 2017-12-28

**Authors:** Elise Paul, Roland Mergl, Ulrich Hegerl

**Affiliations:** 1 Depression Research Centre of the German Depression Foundation, Leipzig, Germany; 2 Department of Psychiatry and Psychotherapy, University Hospital Leipzig, Leipzig, Germany; 3 European Alliance against Depression, Leipzig, Germany; Karolinska Institutet, SWEDEN

## Abstract

Suicide rates in Germany consistently decreased from 1991 to 2006, but this trend was reversed in 2007. Underlying this reversal were large increases in suicides due to gassing in females and in being overrun in males. During a similar time period (2005–2013), Asian and some Western countries have also observed abrupt increases in suicides due to certain gasses, and the availability of “how-to” information on the Internet about these painless methods of suicide is thought to play a role in their increased use. This study used data from the Federal Statistics Office of Germany to examine current trends in overall suicide mortality in Germany (2007–2015) as well by age, gender, and suicide methods. Also assessed was whether suicides via newly emergent methods are associated with the frequency of corresponding Internet searches using data from Google Trends. Joinpoint regression analyses indicated significant increases in the overall suicide rate (Average Annual Percentage Change (AAPC) = 2.37%) for females, but not males. The largest annual increases were observed in gassing self-intoxication suicides (AAPC = 13.93%), the majority of which involved carbon monoxide. The increase in gassing suicides was larger in females (500%), compared to males (164%). The frequency of suicides by gassing was significantly associated with Internet searches for “carbon monoxide poisoning” for both male and female subgroups, independent of age group. This study provides the updated suicide surveillance data that are necessary for suicide prevention activities. Results are congruent with the recent abrupt rises in carbon monoxide suicides in other countries.

## Introduction

In Germany, the rate of overall suicides steadily decreased from 14.19 per 100,000 in 1998 to 11.86 per 100,000 in 2006, but this decline ended and was even slightly reversed from 2007 to 2010 [1]. Underlying this unfavorable change were increases in “intentional self-poisoning by exposure to other gases and vapors than organic solvents and halogenated hydrocarbons and their vapors” (International Classification of Diseases, ICD-10, X67) especially in the young (ages 25 and under) and among females, and in “jumping or lying before moving object”, the majority of which are railway suicides (“being overrun”) in males [1]. While Koburger and colleagues [2] elaborated and attributed the latter finding to the widely publicized railway suicide of German football player Robert Enke, the former trend in gassing suicides remained unexplained.

Since then, other countries have observed large increases in suicides due to certain gasses. The end of the 20^th^ century and the early 2000s were marked by rapid increases in suicide by charcoal burning in Hong Kong and Japan [3,4]. These increases represented changes as large as from 1% to 25% and 30% within a decade [4–6]. More recently, suicides by charcoal burning in Hong Kong and Japan have decreased and this reversal has coincided with substantial increases in the number of suicides due to helium gassing [3,7]. A similar trend (2005–2012) was observed in 16 U.S. states; where helium suicides more than doubled and carbon monoxide suicides declined [8]. The numbers of suicide by helium asphyxiation have also recently risen in Australia and Sweden [9]. During a comparable period (2001–2011) in England and Wales, there was a 17-fold increase in suicide deaths by helium and also large increases in suicide by carbon monoxide poisoning due to charcoal burning [10].

The Internet is thought to play a role in the rapid spread of “how to” information on these highly lethal intoxication methods. As of 2014, an estimated 54% of English-language websites resulting from suicide-related search terms provide factual, step by step “how to” information on high-lethality suicide methods [11]. The availability of such information has tripled since 2007 [11]. The rapid increase in suicides by charcoal-burning is also thought to be related to media portrayals of this method as a painless, peaceful, and effective [12]. In one study, the majority (87%) of individuals who had survived suicide attempt by burning charcoal indicated that the media had influenced their choice of method [13]. It appears that “how to” information is indeed used by people researching and seeking to perfect suicide methods [11,14].

Ecological studies have found mixed support that Internet searches for suicide-related terms correspond to increased rates of suicides [15–17]. Some findings indicate that trends in Internet search activity for specific suicide methods such as gassing are especially associated with suicide rates among those in their 20s and 30s [16]. Others find that more generic suicide-related search terms are inversely[18] or positively related to overall suicide rates [15,17,19]. However, studies investigating associations with rates of method-specific suicides and Internet activity in Western countries are lacking.

The overall goal of this paper is to examine whether there have been changes in specific suicide methods and demographics during the study period 2007–2015. Our first hypothesis is that suicide methods involving self-intoxications due to gassing have continued to increase since the time of the last such report (2007) through the year 2015 [1]. Second, we hypothesize that young individuals (under age 25) and females will have experienced large increases in this suicide method. Third, we expect that increases in suicides by gassing intoxication methods will be associated with Internet search activity for related terms.

## Materials and methods

### Data

#### Suicides

Yearly mortality data on all suicide deaths in Germany during the years 1998 to 2015 were obtained from the Federal Statistical Office of Germany [20]. We chose 1998 as the first year of the study period because it was the first year in which Germany implemented the current version of the International Classification of Diseases, ICD-10 [21]. The primary outcome variable was the crude rate of suicides in Germany from 1998 to 2015. To characterize trends in suicide rates for different population subgroups, the following independent variables were calculated: gender (male vs. female) and age (under 25 years; 25–44 years; 45–64; and 65 years and older).

Suicide methods were based on ICD-10 codes and organized as follows: self-poisoning by psychotropic drugs (X61-X63); self-poisoning by other drugs (X60, X64); self-poisoning due to gasses (X67); self-poisoning by other means (X65, X66, X68, and X69); hanging, strangling or suffocation (X70); drowning or submersion (X71); suicide by firearms (X72-X74); Stabbing with a sharp instrument (X78); jumping from high places (X80); being overrun (X81); and other suicide methods (X75-X77, X79, X82-X84). The identity of the specific gasses used in X67 suicide deaths were obtained from ICD-10 Chapter 19 codes T48.7 (“poisoning by other and unspecified agents primarily acting on the respiratory system—predominantly helium poisoning”), T58 (“toxic effect of carbon monoxide”), and T59.6 (“toxic effect of hydrogen sulphide”).

#### Google searches

We obtained data on the frequency of Google searches made for the terms “depression”, “selbstmord” (synonym of “Suizid”; in English: suicide), “suizid”, “selbstmord methode”, “selbstmord Holzkohle” (in English: suicide by charcoal burning), “exit bag”, and “Kohlenmonoxid vergiftung” (in English: carbon monoxide intoxication) from the Google Trends (http://www.trends.google.com) website. Data were restricted to Germany and from January 1, 2007 through December 31, 2015. The absolute number of searches for each term is not given by Google Trends but is rescaled to a value between 0 and 100. We relied on Google, rather than other search engines, because Google is the most popular search engine in Germany, with approximately 87% and 98% of the market shares for desktop and mobile searches, respectively [22]. Searches for suicide-related terms in Google also return a greater proportion of websites dedicated exclusively to the topic of suicide (30% in contrast to 20.8% among Yahoo, Bing and Ask combined [11]. Although a greater proportion of young and middle aged adults than older adults use the Internet daily, the majority of individuals in the latter group had used the internet at least once in the past three months at the beginning of the study period in 2007 (72%) and at the end (year 2015) (96%) [23].

### Data analysis

To analyze crude German suicide rates between 1998 and 2015, the Joinpoint Regression Program version 4.4.0.0 [24] was used. The independent variable was "year" (1998–2015) and the dependent variable was a count variable of the annual number of suicides in Germany. The yearly German population was selected as the population variable. As heteroscedastic error option, Poisson variance was chosen. The selected regression formula was as follows: ln(y) = a + bx + ln(pop) (with y = the number of suicides, x = the year of suicide and pop = the population number per year). In the next step, the Annual Percentage Change (APC) with the corresponding 95% confidence interval (CI) was estimated for each suicide trend. Tests of parallelism were conducted to compare the mean joinpoint functions between different groups (e.g. males versus females). In these tests, the null hypothesis was that the two groups did not share a common slope.

Next, we explored the possibility that increases in specific suicide methods would be positively associated with trends in Google searches for related terms. To test this, we first converted the frequencies of each search term to z-scores which have a mean of zero and standard deviation of one. We computed Pearson correlations between each of the Google search terms and the number of annual suicides in Germany. To account for possible gender and age confounding in these analyses, correlations were conducted between Google search frequencies and the number of suicides for all suicide methods and gassing suicides (X67) stratified by gender and age. All statistical tests were two-tailed and interpreted at *p* < 0.05. This research was approved by the Leipzig University ethics committee and is therefore in accordance with the Declaration of Helsinki and its later amendments.

## Results

### Overall trends in suicide rates in Germany (1998–2015)

Joinpoint regression analyses were conducted on overall crude suicide rates in Germany during the time span 1998–2015 and revealed 3 joinpoints. The results for 1998–2007 extend what was previously reported [1]. Specifically, the downward trends from 1998–2007 stopped from 2007 until 2011 (Annual Percentage Change (APC) = 2.37; 95% CI: -0.12–4.92; *p* = 0.06), and from 2011 to 2015 there was a slightly negative but non-significant trend (APC = -0.12; 95% CI: -1.64–1.43; *p* = 0.86). Analyses of suicide rates by gender indicated that from 2007 to 2015 there has been a significant increase of suicides in females (APC = 1.70; 95% CI: 0.91–2.50; *p* < 0.01), but not in males. During the same period, two joinpoints were detected for males, neither of which reached statistical significance (2007–2011: APC = 2.78; 95% CI: -0.89–6.58; *p* = 0.12; and 2011–2015: APC = -0.86; 95% CI: -3.09–1.43; *p* = 0.40). Thus, underlying the abrupt reversal in the overall decline in suicides in Germany was a significant increase in female suicides that started in 2007.

When annual overall suicide rates were examined by age and gender, the only age-gender groups with significantly changing annual overall suicide rates were females ages 25–44 (AAPC = 1.19; 95% CI: 0.38–2.00; *p* = 0.01) and middle age females (ages 45–64) (AAPC = 2.27; 95% CI: 1.52–3.03; *p* < 0.01). Trends in suicide rates for individuals under age 25, older adults (ages 65 and up), and males did not significantly change.

### Trends in suicide mortality by specific suicide methods (2007–2015)

Next, we investigated whether there were changes in specific suicide methods underlying the reversal of the decrease in suicides. Of the 11 categories of suicide methods examined, there were no significant changes in the following: self-poisoning by psychotropic drugs (X61-X63), self-poisoning by other drugs (X60, X64), self-poisoning by other means (X65, X66, X68, X69), hanging/suffocation (X70), drowning/submersion (X71), and firearms (X72-X74). Suicides by stabbing with a sharp instrument (X78) (AAPC = 2.29; 95% CI: 0.95–3.65), jumping from a high place (X80) (AAPC = 1.64; 95% CI: 0.10–3.20), and other methods (X75-77, X79, X82-84) (AAPC = 3.05; 95% CI: 0.78–5.37) all significantly increased. Suicide by being overrun (X81), of which most are likely railway suicides, increased from 2007–2010 (APC = 11.41; 95% CI: 5.35–17.81) and then decreased from 2010–2015 (APC = -3.99; 95% CI: -6.27- -1.66). In support of our first hypothesis, the increase in self-poisonings due to gasses (ICD-10) was especially pronounced during the study period (2007–2015) (AAPC = 13.93; 95% CI: 9.35 to 18.71; *p* < 0.001). Demographics and specific gasses underlying this large abrupt increase are elaborated in the next section.

### Suicides by self-poisoning due to gasses (2007–2015)

Our second hypothesis was that young adults and adolescents (under age 25) and females would show especially large increases in gassing suicides (X67). The change in suicide rate by exposure to other gases and vapors (X67) was significantly more pronounced for females (AAPC = 23.29; 95% CI: 16.19 to 30.82; *p* < 0.001) compared to males (AAPC = 12.44; 95% CI: 7.90 to 17.17; *p* < 0.001) (test of parallelism: *p* < 0.001). In raw numbers, these differences corresponded to an increase in the number of gassing suicides in females of 500% from 2007 to 2015, and in males of 165% (Table 1). Young females (under 25 years of age) also had substantial increases in the number of suicides by gassing (+450%), but the increase was highest among women ages 25–44 years (+833%). In males, the increase in gas suicides was highest among the young (ages < 25) (+287.5%). The majority of gassing suicides were due to carbon monoxide (X67_T58) at the beginning (2007; 77.7%) and at the end (2015; 85.7%) of the study period. Suicides due to helium poisoning and hydrogen sulfide were infrequent throughout the study period (fewer than 2 cases per year). Our second hypothesis was therefore confirmed; both the young and females had the largest increases in gassing suicides.

**Table 1:** Demographic distribution of gassing suicides (X67) in Germany (2007–2015).

	Gases and vapors (X67)		
	2007	2015	% Δ
	*n* = 184	*n* = 537	191.9%
	% (*n*)		
Gender			
Females	(15)	(90)	500%
Males	(169)	(447)	164%
Age			
< 25	(10)	(42)	320%
25–44	(46)	(133)	189%
45–64	(109)	(298)	173%
65 +	(19)	(64)	237%
Gender and age			
Females < 25	(2)	(11)	450%
Females 25–44	(3)	(28)	833%
Females 45–64	(8)	(40)	400%
Females 65 +	(2)	(11)	450%
Males < 25	(8)	(31)	288%
Males 25–44	(43)	(105)	144%
Males 45–64	(101)	(258)	155%
Males 65 +	(17)	(53)	212%

### Associations of internet searches with suicide mortality

Our third hypothesis was that increases in gassing suicides (X67) would be positively correlated with the z-standardized frequency of Google searches for related terms. Results are presented in Table 2. The pattern of results indicates that the most consistent and the largest correlations were between Google searches for “depression” and “carbon monoxide poisoning” and gassing suicides in males and females in each age group. In contrast, Google searches for “suicide by charcoal burning” did not correlate with gassing suicides in any of the age-gender subgroups. Only correlations with Google searches for “exit bag”, a method of gassing suicide involving helium, argon, or nitrate, and gassing suicides in young males (< 25 years) and older women (> 65 years) were statistically significant. This pattern of results is partially congruent with our hypothesis and with our finding that the majority of the gassing suicides involved carbon monoxide.

**Table 2:** Pearson correlations between annual frequencies of Google searches with suicides in Germany, 2007–2015.

	depression	suicide (selbstmord)	suicide (suizid)	suicide methods	suicide by charcoal burning	exit bag	carbon monoxide poisoning
Gassing suicides, females (< 25 years)	0.80**	0.57	0.76*	-0.64	0.20	0.30	0.75*
Gassing suicides, males (< 25 years)	0.73*	0.72*	0.55	-0.63	0.53	0.67*	0.89**
Gassing suicides, females (25–44 years)	0.86**	0.53	0.72*	-0.24	0.12	0.32	0.82**
Gassing suicides, males (25–44 years)	0.68*	0.58	0.54	-0.79*	0.54	0.66	0.75*
Gassing suicides, females (45–64 years)	0.99***	0.57	0.87**	-0.25	-0.11	0.22	0.82**
Gassing suicides, males (45–64 years)	0.96***	0.61	0.85**	-0.31	0.03	0.32	0.86**
Gassing suicides, females (65+ years)	0.75*	0.76*	0.54	-0.42	0.48	0.70*	0.93***
Gassing suicides, males (65+ years)	0.91***	0.54	0.74*	-0.49	0.18	0.47	0.81**

* *p* ≤ 0.05

** *p* ≤ 0.01

*** *p* ≤ 0.001.

Gassing suicides = ICD -10 X67.

## Discussion

The overall goal of this study was to investigate current time trends in suicide rates in Germany from 2007 to 2015. Our analyses indicated that the leveling off of the overall suicide rate which was observed in 2006 [1] continued through 2015. While the rate of overall female suicides has steadily increased, the rate of male suicides has not. Time trend analyses confirmed our first hypothesis that underlying the sudden reversal of the decrease in suicides were large increases in self-poisonings due to gassing. In terms of the crude number of gas suicides, the increase was nearly 200% (191.85%) by 2015 compared to 2007. In contrast, the significant increase of suicides in Germany starting from 2007 no longer coincided with increases of suicides by the method “being overrun”. This could point to an attenuation of the increase in railway suicides after the death of Robert Enke which had been present in the two-year period after this tragic event in 2009 [2]. Unemployment and sudden changes in income following the 2008 financial crisis are also factors which could have contributed to these rises in suicides [25].

The alarming trend in gassing suicides has also been observed in Sweden and Australia [9], England and Wales [10], and some US regions but not others [26,27], where carbon monoxide was also the predominant gas used [8,10]. The abrupt changes in suicides by gassing, predominantly carbon monoxide, also correspond to observations in the early 2000s in Hong Kong and Taiwan [3], and contrast with 16 US states, where overall suicide gassing rates remained flat during a similar period (2005–2012) [8]. However, underlying these non-increasing trends in overall gas suicides were substantial increases in suicides due to helium gas [3,8].

In the current study, suicides by other gases such as helium and hydrogen sulfide which have recently increased elsewhere [8,10], were too infrequent to separately identify trends in the current study. While carbon monoxide poisoning made up the majority of the gassing suicides, it could be that the gasses used in the remainder of the gassing suicides were classified as unknown, and could therefore be helium, which is difficult to detect post-mortem. Even when toxicology tests are performed, traces of inert gasses such as helium are either not present or present in very small amounts [28]. The numbers of helium suicides in the present study could therefore have been undercounted.

The increase we observed in suicide by gassing was apparent in both genders, but was significantly more pronounced in females, which is in line with our second hypothesis. The number of female suicides by gassing increased by 500% from 2007 to 2015. The increase in suicides due to gassing in females in Germany corresponds to female preferences for self-intoxication methods and a greater proportion of non-fatal compared to fatal self-intoxication suicidal acts [29]. The high numbers of suicidal acts using intoxication highlight the fact that the availability of information on lethal intoxication methods using commonly available materials such as charcoal will have a pronounced effect on suicide rates, especially for females.

More fine-grained analysis also indicated that young (ages < 25 years) males and young (ages < 25 and ages 25–44 years) females had especially marked changes in suicides by gassing. Our findings are partially congruent with the young and male characteristics of what could be “early adopters” of gassing suicide methods [3,10]. A US study provided descriptive data on gender and found the male: female ratio for helium suicides to be very similar to the national trend [26], although no statistical tests were performed. Researchers from other Western [10,30] and Asian countries [3] have found males and younger individuals to be overrepresented in helium suicides. Helium suicides in Australia increased 163% over similar time periods and suicide decedents ages 20–29 and males were overrepresented [9]. Although research regarding whether gender increases the risk of suicide by gassing is mixed, it does appear that the young are particularly vulnerable.

### Internet search activity and suicides

The sudden increase in carbon monoxide suicides could be explained by the increased availability of suicide-related information in the Internet. We had hypothesized that increases in suicides by gassing methods would be significantly associated with Internet search activity for related terms. In support of this hypothesis, the frequency of suicides by gassing was significantly associated with Internet searches for “carbon monoxide poisoning” for all suicide decedents, independent of age. We did not, however, find significant associations with “suicide by charcoal burning”. It could be that the users of carbon monoxide suicide methods were more likely to use certain search terms rather than others and did in fact use charcoal burning methods. Alternatively, the suicide decedents in this study could have used carbon monoxide methods other than charcoal burning. These findings could indicate an elevated general suicide risk in people searching for online information about carbon monoxide poisoning. In view of the cross-sectional design of our study, it is not possible to derive a causal interpretation of our findings.

It could be that high profile news reports of carbon monoxide suicides have contributed to the recent rapid uptake of this method in Germany, as has been seen in other countries [5,12]. A Google search for news items related to helium suicides during the study period (2007–2015) in Germany corresponded to an increased number of instructions in suicide forums and advise from suicide aid organisations like Exit and Dignitas [31]. News reports for only one celebrity suicide by using helium were documented for Ilya Zhitomirskiy (1989–2011), one of the developers of the open-source software "Diaspora". In contrast, there were several publicized reports of suicides using carbon monoxide gassing methods during the time period examined in the current study. In 2010 in the German town of Kürten, three young (age 20 years) friends died by self-poisoning by burnt gas from a barbecue in a closed room. In August 2011 three more young women (between 16 and 19 years) died by self-poisoning using a charcoal grill.

Additionally, two celebrity suicides which occurred during the study period are also of interest. The US singer Brad Delp (from the rock band Boston) died by suicide when he burnt two barbecues in a closed bathroom. In 2012, Claudia Börner (1979–2012), a model known from a German cooking TV show who had been a victim of cyber mobbing died by suicide using the same method. While we obtained these news reports via Google with specific search terms (carbon monoxide poisoning), it could be that vulnerable individuals were first exposed to these suicide methods through news reports, and then later used specific search terms (e.g. “carbon monoxide poisoning”) to obtain “how-to” information on the method when they were feeling suicidal.

### Limitations

Our analyses relied on the ICD-10 classification system and were therefore unable to determine the source of the gasses that were involved. Determining the source of the gasses used would have provided important information for means restriction efforts [32]. Second, our analyses with Google search data are limited to demonstrating association at the population level, but not temporal precedence. Correspondingly, while several high-profile news reports of gassing suicides took place during the study period, we could not determine whether exposure to these stories had influenced this recent uptake in gassing suicides. In order to demonstrate that the aforementioned media reports about suicides with a certain method led to copycat suicides, we would need a much narrower time frame regarding suicides with this method. Further, individual level and not ecological data are needed to more firmly establish that suicidal persons in Germany were indeed utilizing material found online to inform their choice of suicide method. We were also unable to include data on other suicide risk factors aside from gender and age because of German data protection laws.

### Implications

This research underscores the importance of establishing suicide surveillance systems to identify and prevent the spread of novel suicide methods. Our findings document rising rates of suicide in females, particularly due to self-poisoning by gasses, and indicate carbon monoxide poisoning specifically. The systematic inclusion of information on the source of specific gasses in surveillance will facilitate prevention efforts. Close involvement of the media to discourage detailed reporting of gassing suicide deaths may also be useful in preventing the spread and appeal of these painless suicide methods [33].

### Conclusions

The findings in this study are in line with what has recently been observed regarding abrupt increases in carbon monoxide gassing in several other countries. These increases in self-poisoning suicides by gasses were inconsistently related to Internet searches for related terms. Together, these results underscore the importance of ongoing systematic surveillance not only of suicide methods, but of forums such as the Internet that may provide suicidal individuals with information on suicide methods.

## References

[1] Hegerl U, Mergl R, Doganay G, Reschke K, Rummel-Kluge C. Why Has the Continuous Decline in German Suicide Rates Stopped in 2007? PLOS ONE. 2013;8: e71589 doi: 10.1371/journal.pone.0071589 23967225 10.1371/journal.pone.0071589 PMC3743810

[2] Koburger N, Mergl R, Rummel-Kluge C, Ibelshäuser A, Meise U, Postuvan V, et al Celebrity suicide on the railway network: Can one case trigger international effects? J Affect Disord. 2015;185: 38–46. doi: 10.1016/j.jad.2015.06.037 26143403 10.1016/j.jad.2015.06.037

[3] Chang S-S, Cheng Q, Lee ES, Yip PS. Suicide by gassing in Hong Kong 2005–2013: Emerging trends and characteristics of suicide by helium inhalation. J Affect Disord. 2016;192: 162–166. doi: 10.1016/j.jad.2015.12.026 26724695 10.1016/j.jad.2015.12.026

[4] Yoshioka E, Hanley SJ, Kawanishi Y, Saijo Y. Epidemic of charcoal burning suicide in Japan. Br J Psychiatry. 2014;204: 274–282. doi: 10.1192/bjp.bp.113.135392 24434075 10.1192/bjp.bp.113.135392

[5] Chen Y-Y, Yip PSF, Chan CH, Fu K-W, Chang S-S, Lee WJ, et al The impact of a celebrity’s suicide on the introduction and establishment of a new method of suicide in South Korea. Arch Suicide Res. 2014;18: 221–226. doi: 10.1080/13811118.2013.824840 24620837 10.1080/13811118.2013.824840

[6] Pan Y-J, Liao S-C, Lee M-B. Suicide by charcoal burning in Taiwan, 1995–2006. J Affect Disord. 2010;120: 254–257. doi: 10.1016/j.jad.2009.04.003 19410296 10.1016/j.jad.2009.04.003

[7] Yoshioka E, Hanley SJ, Kawanishi Y, Saijo Y. Time trends in method-specific suicide rates in Japan, 1990–2011. Epidemiol Psychiatr Sci. 2016;25: 58–68. doi: 10.1017/S2045796014000675 25373686 10.1017/S2045796014000675 PMC6998669

[8] Azrael D, Mukamal A, Cohen AP, Gunnell D, Barber C, Miller M. Identifying and tracking gas suicides in the US using the National Violent Death Reporting System, 2005–2012. Am J Prev Med. 2016;51: S219–S225. doi: 10.1016/j.amepre.2016.08.006 27745610 10.1016/j.amepre.2016.08.006

[9] Austin A, Winskog C, van den Heuvel C, Byard RW. Recent trends in suicides utilizing helium. J Forensic Sci. 2011;56: 649–651. doi: 10.1111/j.1556-4029.2011.01723.x 21361949 10.1111/j.1556-4029.2011.01723.x

[10] Gunnell D, Coope C, Fearn V, Wells C, Chang S-S, Hawton K, et al Suicide by gases in England and Wales 2001–2011: Evidence of the emergence of new methods of suicide. J Affect Disord. 2015;170: 190–195. doi: 10.1016/j.jad.2014.08.055 25254616 10.1016/j.jad.2014.08.055

[11] Biddle L, Derges J, Mars B, Heron J, Donovan JL, Potokar J, et al Suicide and the Internet: Changes in the accessibility of suicide-related information between 2007 and 2014. J Affect Disord. 2016;190: 370–375. doi: 10.1016/j.jad.2015.10.028 26546772 10.1016/j.jad.2015.10.028

[12] Chen Y-Y, Tsai C-W, Biddle L, Niederkrotenthaler T, Wu KC-C, Gunnell D. Newspaper reporting and the emergence of charcoal burning suicide in Taiwan: A mixed methods approach. J Affect Disord. 2016;193: 355–361. doi: 10.1016/j.jad.2015.12.041 26796236 10.1016/j.jad.2015.12.041

[13] Tsai C-W, Gunnell D, Chou Y-H, Kuo C-J, Lee M-B, Chen Y-Y. Why do people choose charcoal burning as a method of suicide? An interview based study of survivors in Taiwan. J Affect Disord. 2011;131: 402–407. doi: 10.1016/j.jad.2010.12.013 21236495 10.1016/j.jad.2010.12.013

[14] Gunnell D, Derges J, Chang S-S, Biddle L. Searching for suicide methods. Crisis. 2015;36: 325–331. doi: 10.1027/0227-5910/a000326 26502782 10.1027/0227-5910/a000326

[15] Gunn JF, Lester D. Using Google searches on the internet to monitor suicidal behavior. J Affect Disord. 2013;148: 411–412. doi: 10.1016/j.jad.2012.11.004 23182592 10.1016/j.jad.2012.11.004

[16] Hagihara A, Miyazaki S, Abe T. Internet suicide searches and the incidence of suicide in young people in Japan. Eur Arch Psychiatry Clin Neurosci. 2012;262: 39–46. doi: 10.1007/s00406-011-0212-8 21505949 10.1007/s00406-011-0212-8

[17] Kristoufek L, Moat HS, Preis T. Estimating suicide occurrence statistics using Google Trends. EPJ Data Sci. 2016;5: 32. 10.1140/epjds/s13688-016-0094-0 PMC7175644 32355600

[18] McCarthy MJ. Internet monitoring of suicide risk in the population. J Affect Disord. 2010;122: 277–279. doi: 10.1016/j.jad.2009.08.015 19748681 10.1016/j.jad.2009.08.015 PMC2847052

[19] Sueki H. Does the volume of Internet searches using suicide-related search terms influence the suicide death rate: Data from 2004 to 2009 in Japan. Psychiatry Clin Neurosci. 2011;65: 392–394. doi: 10.1111/j.1440-1819.2011.02216.x 21569178 10.1111/j.1440-1819.2011.02216.x

[20] Statistisches Bundesamt. Gesundheit Statistik Gesundheitsberichterstattung des Bundes [Internet]. 2017 [cited 2 May 2017]. Available: http://www.gbe-bund.de/gbe10/pkg_isgbe5.prc_isgbe?p_uid=gast&p_aid=0&p_sprache=D

[21] World Health Organization. The ICD-10 classification of mental and behavioural disorders: diagnostic criteria for research [Internet]. Geneva: World Health Organization; 1993 Available: http://www.who.int/iris/handle/10665/37108

[22] Search engines: market share of desktop and mobile search in Germany 2017 | Statistic. In: Statista [Internet]. [cited 26 Sep 2017]. Available: https://www.statista.com/statistics/445974/search-engines-market-share-of-desktop-and-mobile-search-germany/

[23] Database—Eurostat [Internet]. [cited 23 Nov 2017]. Available: http://ec.europa.eu/eurostat/web/digital-economy-and-society/data/database

[24] National Cancer Institute SM and AB Surveillance Research Program. Joinpoint Regression Program Version 4.4.0.0. 2017.

[25] Karanikolos M, Heino P, McKee M, Stuckler D, Legido-Quigley H. Effects of the Global Financial Crisis on Health in High-Income Oecd Countries: A Narrative Review. Int J Health Serv. 2016;46: 208–240. doi: 10.1177/0020731416637160 27076651 10.1177/0020731416637160

[26] Cantrell L, Lucas J. Suicide by non-pharmaceutical poisons in San Diego County. Clin Toxicol. 2014;52: 171–175. 10.3109/15563650.2014.888734 24580055

[27] Hassamal S, Keyser-Marcus L, Crouse Breden E, Hobron K, Bhattachan A, Pandurangi A. A brief analysis of suicide methods and trends in Virginia from 2003 to 2012. BioMed Res Int. 2015;2015. 10.1155/2015/104036 PMC4331312 25705647

[28] Oosting R, van der Hulst R, Peschier L, Verschraagen M. Toxicological findings in three cases of suicidal asphyxiation with helium. Forensic Sci Int. 2015;256: 38–41. doi: 10.1016/j.forsciint.2015.06.028 26298854 10.1016/j.forsciint.2015.06.028

[29] Mergl R, Koburger N, Heinrichs K, Székely A, Tóth MD, Coyne J, et al What are reasons for the large gender differences in the lethality of suicidal acts? An epidemiological analysis in four European countries. PLOS ONE. 2015;10: e0129062 doi: 10.1371/journal.pone.0129062 26147965 10.1371/journal.pone.0129062 PMC4492725

[30] Howard MO, Hall MT, Edwards JD, Vaughn MG, Perron BE, Winecker RE. Suicide by asphyxiation due to helium inhalation. Am J Forensic Med Pathol. 2011;32: 61–70. 21394956 10.1097/paf.0b013e3181ed7a2d

[31] Grassberger M, Krauskopf A. Suicidal asphyxiation with helium: report of three cases. Wien Klin Wochenschr. 2007;119: 323–325. doi: 10.1007/s00508-007-0785-4 17571238 10.1007/s00508-007-0785-4

[32] Yip PS, Caine E, Yousuf S, Chang S-S, Wu KC-C, Chen Y-Y. Means restriction for suicide prevention. The Lancet. 2012;379: 2393–2399. 10.1016/S0140-6736(12)60521-2 PMC6191653 22726520

[33] Yip PS, Cheng Q, Chang S-S, Lee EST, Lai CC, Chen F, et al A Public Health Approach in Responding to the Spread of Helium Suicide in Hong Kong. Crisis. 2017;38: 269–277. doi: 10.1027/0227-5910/a000449 28337929 10.1027/0227-5910/a000449

